# Exploring the relation between people’s theories of intelligence and beliefs about brain development

**DOI:** 10.3389/fpsyg.2015.00921

**Published:** 2015-07-03

**Authors:** Ashley J. Thomas, Barbara W. Sarnecka

**Affiliations:** Sarnecka Cognitive Development Lab, Department of Cognitive Sciences, University of California, Irvine, Irvine, CAUSA

**Keywords:** implicit theories of intelligence, essentialism, folk psychology, naïve biology

## Abstract

A person’s belief about whether intelligence can change (called their implicit theory of intelligence) predicts something about that person’s thinking and behavior. People who believe intelligence is fixed (called entity theorists) attribute failure to traits (i.e., “I failed the test because I’m not smart.”) and tend to be less motivated in school; those who believe intelligence is malleable (called incremental theorists) tend to attribute failure to behavior (i.e., “I failed the test because I didn’t study.”) and are more motivated in school. In previous studies, researchers have characterized participants as either entity or incremental theorists based on their agreement or disagreement with three statements. The present study further explored the theories-of-intelligence (TOI) construct in two ways: first, we asked whether these theories are coherent, in the sense that they show up not only in participants’ responses to the three standard assessment items, but on a broad range of questions about intelligence and the brain. Second, we asked whether these theories are discrete or continuous. In other words, we asked whether people believe one thing or the other (i.e., that intelligence is malleable or fixed), or if there is a continuous range of beliefs (i.e., people believe in malleability to a greater or lesser degree). Study (1) asked participants a range of general questions about the malleability of intelligence and the brain. Study (2) asked participants more specific questions about the brains of a pair of identical twins who were separated at birth. Results showed that TOI are coherent: participants’ responses to the three standard survey items are correlated with their responses to questions about the brain. But the theories are not discrete: although responses to the three standard survey items fell into a bimodal distribution, responses to the broader range of questions fell into a normal distribution suggesting the theories are continuous.

## Introduction

Suppose your 10-years-old daughter comes bouncing through the front door, proudly waving a report card full of A’s. Do you say, (a) “You’re so smart!” or (b) “You worked so hard!”? If you study motivation, you undoubtedly choose (b), because of the many studies showing that praising children for effort (rather than intelligence) encourages them to believe that intelligence is malleable. In the literature, this is called having an ‘incremental’ theory of intelligence, or being an ‘incremental theorist’ (see Dweck, 2000 for review). Incremental theorists see challenging situations as opportunities to learn. They persevere on difficult tasks and attribute their failures to controllable factors (i.e., “I failed because I didn’t study.”). The opposite of an incremental theorist is an ‘entity theorist,’ someone who views intelligence as a fixed entity. Entity theorists see challenging situations as tests of intelligence. They give up sooner than incremental theorists and attribute their failures to fixed traits (i.e., “I failed because I am dumb”; Mueller and Dweck, 1998; Kamins and Dweck, 1999; Dweck, 2000; Blackwell et al., 2007; Gunderson et al., 2013). In other words, a person’s theory of intelligence (specifically, their belief about whether intelligence is a fixed entity or an incrementally developing skill) affects that person’s thinking and behavior in important ways. The present study asks two questions about theories of intelligence (TOI): first, are they coherent? Second, are they discrete or continuous?

### Coherence and Breadth of Theories of Intelligence

Calling these beliefs *theories* implies that they are rich, structured modes of reasoning (e.g., Dweck et al., 1995a; Aronson et al., 2002; Blackwell et al., 2007; Miele and Molden, 2010). But most researchers diagnose participants’ TOI using the same three items: *(1) Your intelligence is something about you that you can’t change very much. (2) You have a certain amount of intelligence and you can’t do much to change it. (3) You can learn new things, but you can’t really change your basic intelligence*. People who agree with these statements are entity theorists; those who disagree are incremental theorists (see Dweck et al., 1995a for review). In other domains, folk theories (also called naïve theories) do appear coherent. This has been shown with folk theories of cosmology (Vosniadou and Brewer, 1992), evolution (Shtulman and Schulz, 2008; Shtulman and Calabi, 2012), matter (Smith, 2007), motion (Caramazza et al., 1981), and physics (Lautrey and Mazens, 2004). In the present study, we wanted to find out whether folk TOI are similarly coherent. If so, then we should expect to find strong correlations between people’s responses to the three standard assessment items and their responses to other questions about the malleability of intelligence and the brain. For example, entity theorists might be expected to believe that a person’s brain is determined more by their genetics than their environment, whereas incremental theorists might believe that a person can change their brain with practice (just as they believe that a person can change their intelligence if they practice).

One reason to expect that people’s theories are coherent comes from the literature on psychological essentialism. Essentialism is a mode of reasoning based on the intuition that natural kinds have underlying ‘essences’ that are responsible for their observable traits. These essences are assumed to be invisible, extremely difficult to change, and biologically based. An example of an essence is IQ: people who essentialize intelligence often imagine IQ as the invisible and unchanging essence that causes a person to succeed or fail at intellectual tasks (Gelman, 2005). Gelman et al. (2007) found that people’s essentialist beliefs about intelligence are coherent. People who believe that intelligence is fixed (i.e., entity theorists) tend to also believe that it is biologically based and a pervasive part of one’s personality. Conversely, people who believe that intelligence are malleable (i.e., incremental theorists) tend to reject essentialist assumptions. Thus, if TOI are coherent, then participants’ responses to the three standard assessment items should correlate with their beliefs about genetics and environmental influences on the developing brain.

In the present study, we asked participants questions about the developing brain. The questions were divided into six thematic categories, chosen to reflect a broad range of possible influences on the brain. In alphabetical order, these categories were: **(1) Brain Basis Traits**: If entity theorists have a coherent and essentialized view of intelligence, they should be more likely than incremental theorists to attribute psychological characteristics to physical properties of the brain. (**2) Environment:** similarly, incremental theorists should assign the environment a greater role in brain development than entity theorists do. **(3) Innateness**: Entity theorists should be more likely than incremental theorists to believe that intellectual characteristics are innate. (**4**) **Learning**: Incremental theorists should be more likely than entity theorists to believe that learning changes the brain. (**5) Practice:** Similarly, incremental theorists should be more willing to believe that practicing changes the brain. (**6) Willful Control:** incremental theorists should be more likely to say that individuals have some control over how their brains develop.

### Are Theories of Intelligence Categorical or Continuous?

Unlike most research using the theories-of-intelligence construct, the analyses by Gelman et al. (2007) suggest that people’s beliefs about intelligence may fall anywhere along a continuous spectrum from very essentialized to not-at-all essentialized, rather than falling into the two distinct groups that are typically discussed in the TOI literature (i.e., entity theorists and incremental theorists). Thus, the second question we ask in the present paper is: are TOI discrete or continuous? In other words, do people tend to hold one theory or the other (i.e., are people *either* entity theorists *or* incremental theorists)? Or do these beliefs lie on a continuum? In the present paper, we explored this in two ways. First, we gave participants the three standard assessment items, but included a neutral answer option so that participants were not forced to choose one side or the other. Second, we looked at the distribution of responses for both the three standard assessment items, and for a broader range of statements about intelligence and brain development. If people naturally fall into two camps in their thinking about intelligence, the distribution of responses should be bimodal, with entity theorists producing one cluster of responses and incremental theorists producing another cluster.

## Study 1

### Materials and Methods

All research activities were overseen and approved by the Institutional Review Board for Human Subjects Research at the University of California – Irvine.

#### Participants

Participants included 220 adults who were paid $1.00 each to complete a survey over the internet. Participants were recruited through the Amazon Mechanical Turk System for Human Intelligence Tasks. The survey included four items meant to check if participants were reading carefully (e.g., “There are consistent differences between the brains of aliens and humans. Please answer ‘disagree.”’) If participants did not answer as directed, their data were excluded; 27 participants’ data were excluded for this reason. Reported age of the remaining 193 participants ranged from 18 to 69 years old (*M* = 33.5); reported genders were male (*n* = 105, 54%), transgender (*n* = 1, 0.05%) and female (*n* = 87, 45%). Participants also indicated their race/ethnicity by choosing from a list: responses included white/Caucasian (*n* = 147, 76.96%); Asian/Pacific Islander (*n* = 17, 8.9%); black/African–American (*n* = 10, 5.24%); Chicano/Latino (*n* = 13, 6.81%) and Native American/Alaska Native (*n* = 2, 1.05%). Two participants (1.05%) declined to report their race/ethnicity. Participants were also asked to indicate the highest level of education they had completed. Answers included post-graduate degree (*n* = 13, 6.74%), some graduate school (*n* = 11, 5.7%); Bachelor’s degree (*n* = 68, 35.23%), Associate’s degree (*n* = 21, 10.88%); some college, no degree (*n* = 53, 27.46%), high school or equivalent (*n* = 25, 12.0%), and less than high school (*n* = 2, 1.04%).

#### Materials and Procedure

The survey included 45 items. These 45 included the four reading-check items and the four demographic questions mentioned above; the remaining 37 items formed the basis for the analysis. The four reading check questions and the remaining 37 items were presented in a random order. The 37 items included the three standard assessment theory-of-intelligence items used in previous studies; eight items on intelligence and essentialism, and 26 items on brain development and plasticity. Each question used a five point Likert scale that was appropriate for the statement. Usually ranging from “Strongly disagree” to “Strongly agree” (See Supplementary Materials for a complete list of questions and possible responses).

##### Standard assessment items for theory of intelligence (three items)

As noted in the introduction, most studies assess participants’ TOI by asking them to agree or disagree with these three statements (Dweck et al., 1995a). We used the same statements, but gave participants a five point Likert scale that ranged from “Strongly disagree” to “Strongly Agree” and included a “neutral” response, instead of a six point Likert scale that is usually used. The three standard questions are:

1. Your intelligence is something about you that you can’t change very much.
2. You have a certain amount of intelligence and you can’t do much to change it.
3. You can learn new things, but you can’t really change your basic intelligence.

##### Essentialism (eight items)

We used these items to explore the essentialism in participants’ thinking about intelligence by asking whether intelligence is biologically based, difficult to change, present at birth and pervasive (Haslam et al., 2006; Gelman et al., 2007). For example, “*Being intelligent has broad ramifications: it influences people’s behavior in a wide variety of situations and in many aspects of their lives.”* (A complete list of survey items appears on the supplemental materials.)

##### The brain (26 items)

We used these items to explore participants’ beliefs about the brain. The statements belonged to six thematic categories (presented here in alphabetical order).

##### Brain basis of traits (seven items)

These items asked whether people with different traits have different brains. For example, “There are consistent differences between the brains of people who are intelligent and the brains of people who are not intelligent.”

##### Environment (three items)

These items asked whether the environment affects brain development. For example, “Depending on environment, a child’s brain will develop in different ways.”

##### Innateness (five items)

These items asked whether the characteristics of people’s brains at birth determine their future abilities. For example, “The characteristics of a person’s brain at birth is the largest determining factor in whether or not that person will be considered a genius later in life.”

##### Learning (five items)

These items asked participants whether learning changes the brain. For example, “There is a lasting change in a person’s brain after he or she learns how to count to ten.”

##### Practice (four items)

These items asked whether practicing a skill changes the brain. For example, “If a person practices speaking a new language, their brain will change as a result.”

##### Willful control (two items)

These items asked whether people have control over their brains. For example, “A person can change their brain if they want to.”

### Results and Discussion

Our overall analytical approach was to compare participant’s theory-of-intelligence scores (as measured by the three standard assessment items) to their responses on the eight Essentialism items and the 26 Brain items. We considered *p*-values under 0.006 to be significant, after a Bonferonni correction for multiple analyses (0.05/8) because we used the theory of intelligence questions in eight separate analyses.

We also looked at the distributions of responses. To avoid making *a priori* assumptions about whether TOI are continuous or discrete, we included both analyses using simple linear regression (which treat theory-of-intelligence as a continuous variable) and one-way ANOVAs (which treat it as a categorical variable). We also wanted to include the ANOVAs in order to make it easier to compare our findings to the rest of the literature, which treats theory-of-intelligence as a categorical variable.

### Standard Assessment for Theory of Intelligence (Three Items)

We started by averaging each participant’s responses to the three standard assessment items to create their theory-of-intelligence score. Mean scores ranged from 1 to 5 (*M* = 2.77, SD = 1.02) with higher scores indicating a stronger belief that intelligence is fixed. There was no correlation between participants’ ages and their theory-of-intelligence scores (*R*^2^ = 0.001, *p* = 0.288); nor was there an effect of gender [*F*(2,190) = 0.708, *p* = 0.494] or race [*F*(6,186) = 0.624, *p* = 0.711]. There was, however, a marginally significant effect of education level [*F*(6,186) = 2.12, *p* = 0.053]. *Post hoc* comparisons showed significant differences between participants who had ‘some college but no degree’ and people with bachelor’s degrees [*t*(112) = 2.864, *p* = 0.005] and also between people who had ‘some college but no degree’ and people who had ‘some graduate school’ [*t*(15) = 2.687, *p*= 0.017]. People who indicated that they had ‘some college but no degree’ saw intelligence as more malleable than those with bachelor’s degrees and those who had some graduate school. There was also a difference between those who had ‘graduated high school or equivalent’ and those with ‘some graduate school’ [*t*(24) = 2.06, *p* = 0.05] where people who had graduated high school saw intelligence as more malleable than those with ‘some graduate school.’ Treating education level as a continuous variable (i.e., from 1 to 7) there was a small but significant correlation between theory of intelligence scores and education level (*R*^2^ = 0.042, *p* < 0.005) where people who were more educated believed intelligence to be more fixed.

#### Sorting into theory-of-intelligence groups

Most studies in the TOI literature use a six-point scale, which includes no neutral response. On that scale, every response is either on the entity side or on the incremental side of the scale. We used a five-point scale so that participants could answer ‘neutral’ if they wanted. Studies using the six-point scale typically sort participants into groups by splitting the scale down the middle: people with mean scores of 1–3 are called incremental theorists; those with mean scores of 4–6 are called entity theorists, and those whose means fall in between 3 and 4 are excluded from the analyses (Dweck et al., 1995b). In order to make our findings easier to compare with the rest of the literature, we also sorted participants into categories (i.e., entity theorists, neutral and incremental theorists) based on their TOI scores. We did this by separating the responses into terciles: participants with mean scores from 1 to 2.332 were labeled *incremental theorists* (*n* = 81); those with scores from 2.333 to 3.666 were labeled *neutral theorists* (*n* = 50); those with scores from 3.667 to 5 were labeled *entity theorists* (*n* = 62).

### Essentialism (Eight Items)

We calculated an essentialism score for each person by averaging over the eight essentialism questions. These scores ranged from 1.5 to 4.63 (*M* = 3.30, SD = 0.52) where higher scores indicate a more essentialized view of intelligence. There was no correlation between people’s age and these scores (*R*^2^ = 0.00, *p* = 0.678) no was there effects of education [*F*(6,186) = 0.81, *p* = 0.565] race [*F*(6,186) = 1.58, *p* = 0.168], or gender [*F*(2,190) = 2.06, *p* = 0.13].

#### Coherence of theories of intelligence with essentialism

**FIGURE 1:**
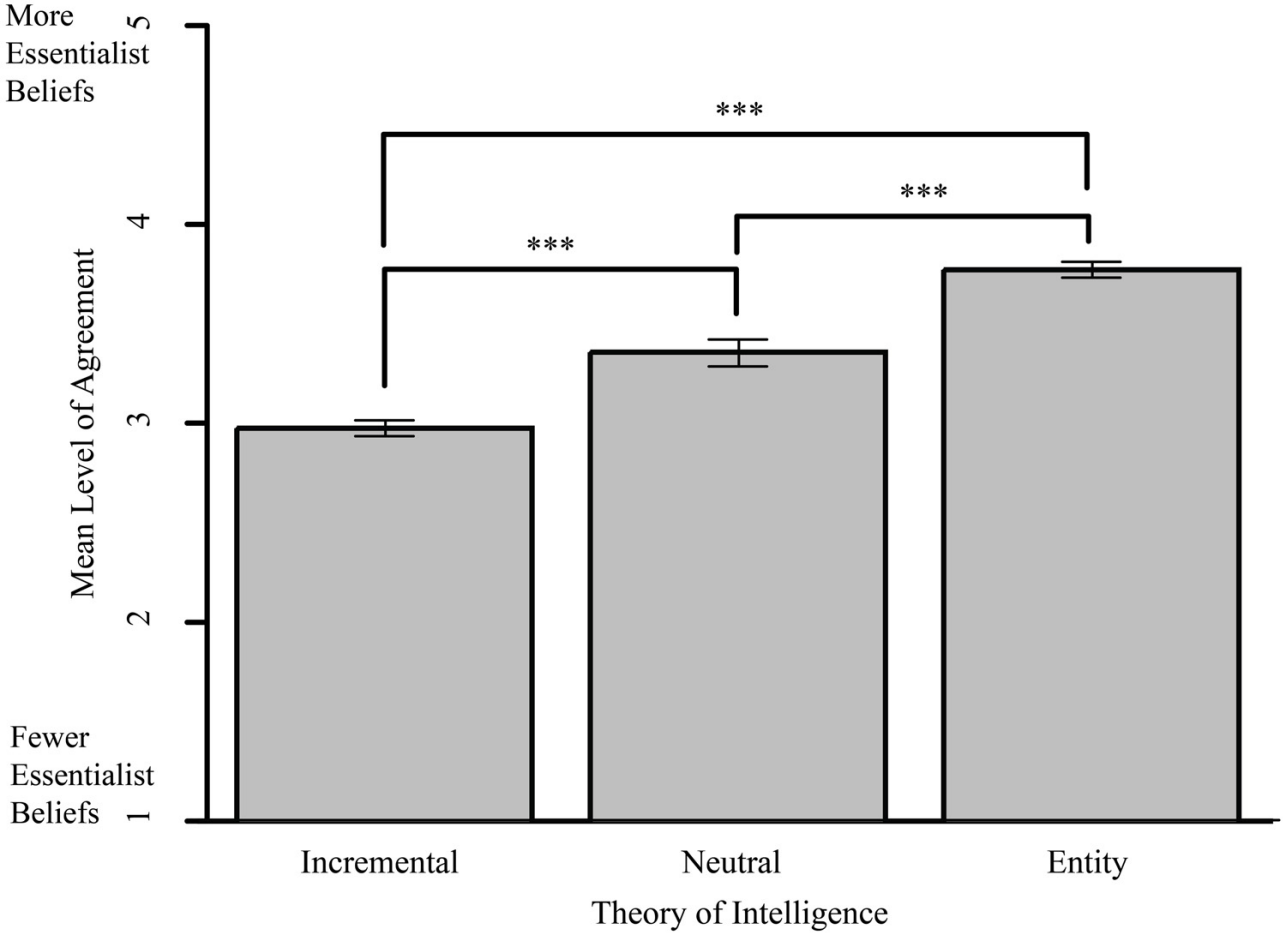
**Essentialism scores by theory of intelligence (TOI).** Mean level of agreement with statements such as “People who have intelligence will tend to display it in a consistent manner, showing it in different situations and with different people,” by participants in the different TOI groups. Error bars indicate SE. ***p < 0.001.

Participants’ TOI scores were correlated with their responses to the essentialism questions. Treating TOI as continuous, we found that the more a person endorsed an ‘entity’ TOI (as measured by the three standard assessment items), the more they saw intelligence as essentialized: that is, biologically based, pervasive, and difficult to change (*R*^2^= 0.40, *p* < 0.001). Treating TOI as categorical, there was a main effect of TOI on essentialism responses [*F*(2,190) = 80.94, *p* < 0.001, η^2^ = 0.460]. Tukey contrasts show that incremental theorists rejected essentialist statements the most, followed by neutral theorists and then entity theorists (entity and incremental [*t*(141) = 13.40, *p* < 0.001] entity and neutral [*t*(92) = 6.94, *p* < 0.001] and neutral and incremental [*t*(106) = 4.59, *p* < 0.001; see **Figure 1**]).

### Questions about the Brain (26 items)

We calculated each person’s beliefs about the brain by averaging across the 26 brain items. The mean scores for these brain questions ranged from 1.96 to 4.36 (*M* = 3.4, SD = 0.329) where higher scores indicate that a person believes the brain is malleable. There was no correlation between people’s age and these scores (*R*^2^ = 0.01, *p* = 0.29) nor was there effects of education [*F*(6,186) = 0.38, *p* = 0.862] race [*F*(6,186) = 0.470, *p* = 0.830], or gender [*F*(2,190) = 2.27, *p* = 0.11].

#### Coherence of theories of intelligence with beliefs about the brain

**FIGURE 2:**
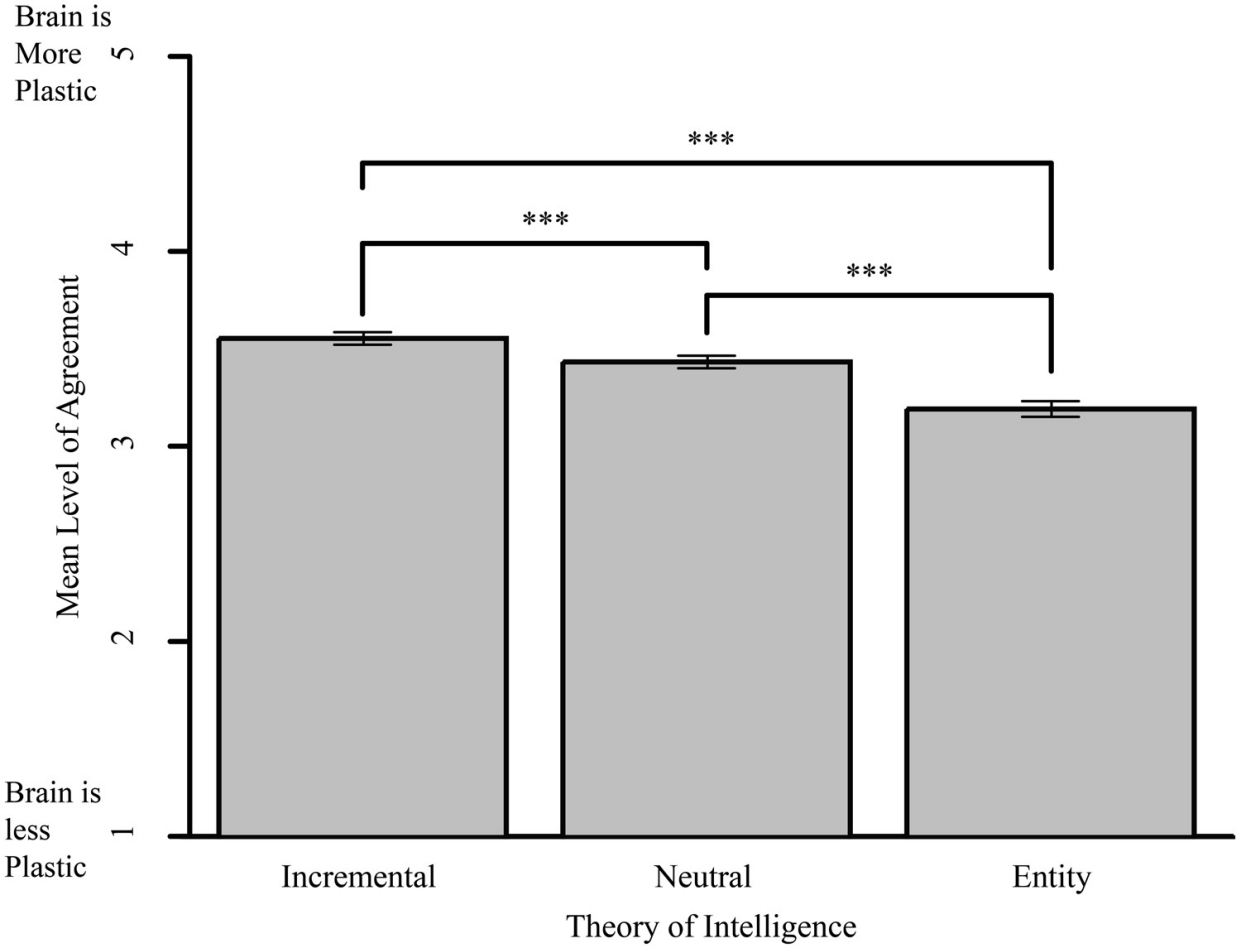
**Theories of intelligence and beliefs about brain plasticity.** Mean level of agreement with statements such as “There is a lasting change in a person’s brain after he or she learns a new language,” by participants in the different TOI groups. Error bars indicate SE. ***p < 0.001.

Participants’ responses to the three standard assessment TOI questions were also consistent with their responses to the brain questions. If we treat TOI as continuous, we can say that the more a person believed intelligence to be changeable, the more they endorsed statements about the brain itself being plastic (*R*^2^ = 0.313, *p* < 0.001). If we treat TOI as discrete categories, a one-way ANOVA showed a main effect of TOI on questions about the brain [*F*(2,190) = 36.11, *p* < 0.001, η^2^ = 0.275]. Tukey contrasts showed significant differences between all three of the TOI categories (entity and incremental [*t*(122) = 7.74, *p* < 0.001] entity and neutral [*t*(104) = 4.185, *p* < 0.001] and neutral and entity [(*t*(57) = 4.6 *p* < 0.001; see **Figure 2**]).

We also looked at the relations between participants’ TOI scores and their responses to the different subtypes of brain items. Below, we report the results for each item subtype in order of effect size, beginning with the largest.

#### Willful control (two items)

There was a strong correlation between TOI scores and responses to the two items about willful control, such as “A person can change their brain if they want to.” (*R*^2^ = 0.30, *p* < 0.001). The more a participant believed that intelligence was malleable, the more they thought that people have control over their brains. Treating TOI as a categorical variable, we found a main effect of TOI group on the Willful Control items [*F*(2,190) = 30.2, *p* < 0.001, η^2^ = 0.241]. Tukey comparisons revealed significant differences between all three groups, where incremental theorists most agreed that people have control over their brains, entity theorists most disagreed with this idea; and neutral theorists fell in between [entity and incremental *t*(131) = 7.72, *p* < 0.001] entity and neutral [*t*(106) = 4.07, *p* < 001] and neutral and incremental [*t*(106) = 3.03, *p* < 0.01].

#### Innateness (five items)

There was a strong correlation between TOI scores and judgments about the innateness of brain characteristics, such as “The characteristics of a person’s brain at birth is the largest determining factor in whether or not that person will be considered a genius later in life.” (*R*^2^ = 0.22, *p* < 0.001). The more a participant believed that intelligence is fixed, the more they believed that a baby’s brain at birth determines his or her future intellectual abilities, including learning how to read, being able to learn calculus and being a genius. Treating TOI as a categorical variable, we found a main effect of TOI group on the Innateness items [*F*(2,189) = 26.31, *p* < 0.001, η^2^ = 0.218]. Tukey comparisons revealed significant differences between all three groups, where entity theorists most endorsed the idea that brain changes are determined by genetics; incremental theorists most disagreed with this idea; and neutral theorists fell in between [incremental and neutral *t*(134) = 7.01, *p* < 0.001; entity and neutral *t*(109) = 4.34, *p* < 001; neutral and incremental *t*(121) = 2.73, *p* < 0.05].

#### Brain basis of traits (seven items)

There was a small but significant correlation between TOI scores and judgments about whether people with different traits have corresponding differences in their brains, such as, “There are consistent differences between the brains of people who are good at math and people who are bad at math” (*R*^2^ = 0.11, *p* < 0.001). The more a participant believed that intelligence is malleable, the more they thought that people with different traits had corresponding brain differences. Treating TOI as a categorical variable, we found a small, but significant effect on these responses [*F*(2,190) = 8.33, *p* < 0.001, η^2^ = 0.081]. Tukey comparisons revealed that the ‘entity’ group differed significantly from the other two groups, in that entity theorists were less likely than the other two groups to believe that traits correspond to brain characteristics [entity and incremental *t*(141) = 3.91, *p* < 0.001; entity and neutral *t*(106) = 3.43, *p* < 0.005; neutral and incremental *t*(126) = 0.652, *p* = 1.0].

#### Environment (three items)

There was a small but significant correlation between TOI scores and judgments about whether the environment plays a role in brain development and (*R*^2^ = 0.087, *p* < 0.001). The more a participant believed that intelligence is malleable, the more they thought that the environment affects brain development. Treating TOI as a categorical variable, we found a small, but significant effect on these responses [*F*(2,190) = 7.27, *p* < 0.001, η^2^ = 0.072]. Tukey comparisons revealed that the ‘entity’ group differed significantly from the other two groups, in that entity theorists were less likely than the other two groups to attribute brain characteristics to the environment [entity and incremental *t*(132) = 3.70, *p* < 0.001; entity and neutral *t*(110) = 2.26, *p* < 0.05; neutral and incremental *t*(116) = 1.375, *p* = 0.172].

#### Practice (four items)

There was a small but significant correlation between TOI scores and beliefs about whether practicing skills changes a person brain (*R*^2^ = 0.05, *p* = 0.002). The more a participant believed that intelligence is malleable, the more they thought that practicing changes the brain. Treating TOI as a categorical variable, we found a marginally significant effect of TOI category on whether participants believed practicing skills changes a person’s brain [*F*(2,190) = 4.79, *p* = 0.009, η^2^ = 0.038] Tukey contrasts revealed a significant difference only between the incremental and the entity theorists [*t*(112) = 2.77, *p* = 0.02].

#### Learning (five items)

The group of survey items least correlated with TOI scores were those asking whether learning is associated with changes in the brain. Most participants agreed that it is: for example, 72% of participants agreed or strongly agreed with the statement, “Learning is due to modifications in the brain”; and 90% agreed or strongly agreed that, “There is a lasting change in the brain when someone learns a new skill.” Using the Bonferroni-adjusted alpha level of 0.006, we found that the correlation between TOI scores and responses to these questions did not reach statistical significance (*R*^2^ = 0.036, *p*= 0.008). Treating TOI as a categorical variable, the main effect of TOI category on responses to these survey items also was not significant [*F*(2,190) = 3.412, *p* = 0.035, η^2^ = 0.035].

### Are Theories of Intelligence Categorical or Continuous?

**FIGURE 3:**
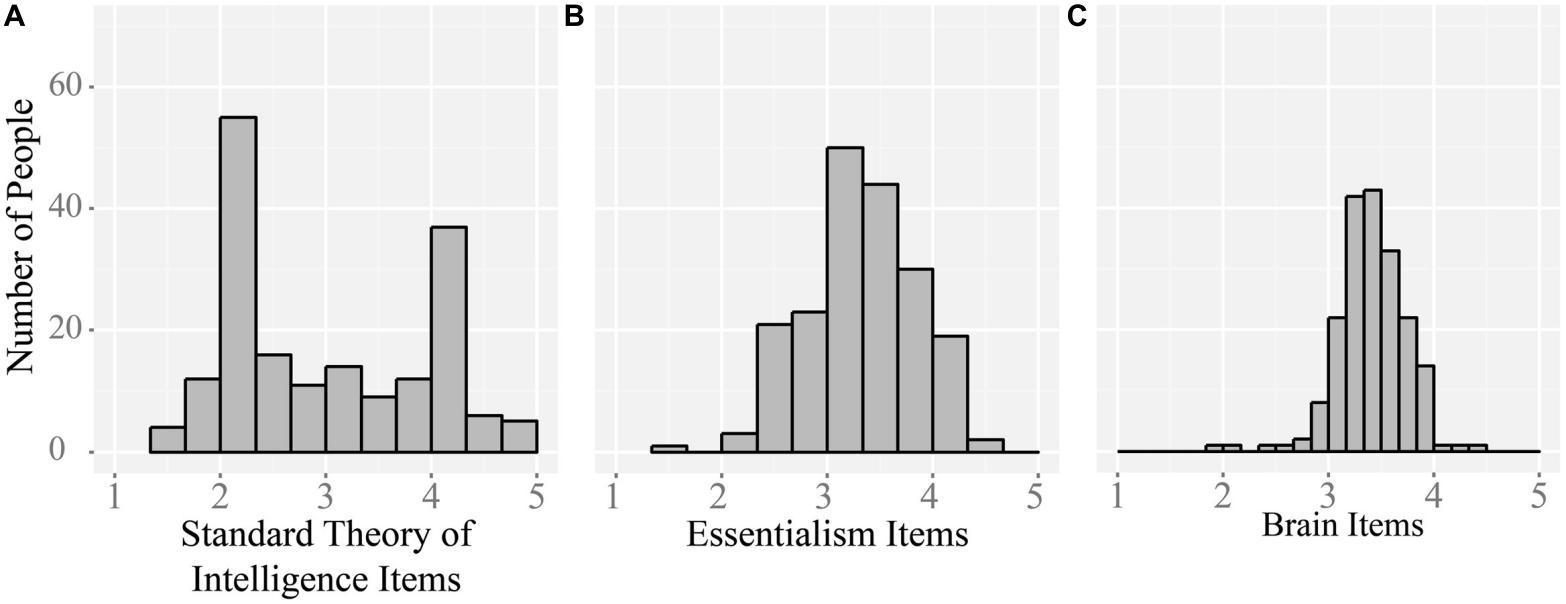
**Distributions.** Histograms of averaged responses for the three standard TOI assessment items **(A)**; the eight essentialism items **(B)** and the 26 brain items **(C)**. Only **(A)** shows a strongly bimodal distribution, indicating that theories of intelligence only appear categorical when assessed using the three standard questions. When participants’ beliefs are assessed using a broader set of questions (as in **B,C**) theories of intelligence appear to vary along a continuous dimension from very entity-based/essentialist to very incremental/non-essentialist.

The answer to this question was different for the three standard TOI questions than for the questions about essentialism and questions about the brain (see **Figure 3**). Although responses to the standard assessment items did form a bimodal distribution (indicating that people *either* saw intelligence as fixed *or* as malleable), responses to the broader sets of questions about essentialism and the brain were relatively normally distributed. This difference can be formally described using a Kolmogorov–Smirnov test for normality. In this test, a distribution is normal if the *p*-value is above 0.05. The distribution of the TOI questions are not normally distributed (*D* = 0.1934, *p* < 0.001, *M* = 2.77, SD = 1.025), while the distributions of the essentialism questions (*D* = 0.0788, *p* = 0.1819, *M* = 3.03, SD = 0.523) and the questions about the brain (*D* = 0.071, *p* = 0.2913, *M* = 3.41, SD = 0.329) are normally distributed.

We interpret these data as showing that there is a range of opinions about whether intelligence is malleable or not, and range of related opinions about factors that influence brain development. People may fall anywhere on a continuum from believing very strongly that intelligence is malleable to believing very strongly that intelligence is fixed. The three standard assessment items allow researchers to distill this continuous variation into two relatively neat categories (entity theorists and incremental theorists) into which most participants can be sorted.

In Study 1, we set out to answer two questions about TOI. The first was whether these theories are coherent, in the sense that they show up not only in responses to the three standard assessment items, but to a wide range of questions about essentialism and the brain. We concluded that the theories are indeed coherent: people’s responses on the three standard items were strongly correlated with their responses to the eight novel essentialism items and 26 novel brain items. For example, the more a person believed endorsed the entity TOI, the more they believed that brain development is determined by genetics instead of environment, and the less they agreed that a person could change their brain if they want to. Similarly, the more a person believed that intelligence can change, the more they believed that practice and the environment have important effects on brain development. These correlations suggest that TOI are not merely an artifact of the three standard survey items typically used to assess them. They are rich, structured modes of reasoning deserving of the word ‘theory.’

The second question we set out to answer was whether the TOI are categorical or continuous. Here, responses to the broader range of questions suggested that these theories exist on a continuum from very essentialist/entity-based to very non-essentialist/incremental. Although participants’ responses to three standard assessment items did form a bimodal distribution (suggesting that most people fall into either the entity category or the incremental category), this was not the case for the broader range of questions. Participants’ answers to the eight essentialism questions and the 26 brain questions were relatively normally distributed, suggesting that most people believe (at least to some degree) in both fixedness *and* malleability.

## Study 2

Study 1 asked people to reason about abstract statements, but it is possible for people to hold a coherent set of abstract beliefs, and not apply those beliefs when reasoning about specific situations. In Study 2, we asked people to make judgments about the genetic and environmental effects on the brain in a concrete scenario.

### Materials and Methods

All research activities were overseen and approved by the Institutional Review Board for Human Subjects Research at the University of California – Irvine.

#### Participants

Participants included 68 people were paid $1.00 each to complete a survey over the internet. Participants were recruited from the Amazon Mechanical Turk system for human intelligence tasks. Four participants were excluded because they incorrectly answered questions that were meant to check whether participants were reading the questions. According to their self-reports, the remaining participants were 21–76 years old (mean = 39) and included 24 males (37.5%) and 40 females (62.5%). Races reported included 56 Caucasian (87.5%); one Black/African–American (1.5%), three Other/Multi Racial (4.7%), two Asian/Pacific Islander (3.1%). Two participants declined to state their race (3.1%). Participants were also asked to indicate the highest level of education they had completed. Answers included post-graduate degree (*n* = 8, 12.7%), some graduate school (*n* = 0, 0%); Bachelor’s degree (*n* = 24, 38.1%), Associate’s degree (*n* = 6, 9.52%); some college, no degree (*n* = 13, 20.6%), high school or equivalent (*n* = 12, 19.0%), and less than high school (*n* = 0, 0%).

#### Materials and Procedures

All participants started out by reading the following vignette:

Scott and Paul are identical twins who were separated at birth when they were adopted by different families. Both Scott and Paul were adopted into families that had sons who were their same age.

Scott and his adoptive brother Dan attended the same schools their entire lives, all of which were known for their academic rigor. The importance of academics was stressed in the household. Both Scott and Dan were expected to do well in school. Scott’s adoptive parents provided tutors, homework help, or any other academic resource necessary to help Scott and Dan do well in school. Scott was very motivated to be a good student to please his adoptive parents.

Paul and his adoptive brother Luke also attended the same schools their entire lives, all of which were known for their poor academics. The importance of academics was not stressed in the household. Instead, Paul’s adoptive parents stressed the importance of athletics. Both Paul and Luke were expected to play sports and excel as athletes. Paul’s adoptive parents provided coaches, trainers, or any other athletic resource necessary to help Paul and Luke do well in sports. Paul was very motivated to be a good athlete to please his adoptive parents.

After reading the vignette, participants answered 18 survey items: four questions to check whether the participants were reading the questions, three standard TOI assessment items, the eight essentialism questions used in Survey 1, and three questions asking them to predict how similar the twins’ brains would be to one another, and how similar the twins’ brains would be to their adoptive brothers. For example, “At age 25, how different are Scott and Paul’s brains? (Scott and Paul are the identical twins and have never met.)” The 18 questions were presented in random order. The three standard assessment items and eight essentialism items used a five-point Likert scale with response choices ranging from “Strongly disagree” to “Strongly agree.” The three questions about the twins used a seven-point Likert scale with response choices from “Their brains are entirely different (meaning as different as two human brains can be)” to “Their brains are identical.” We used a seven-point Likert scale for the twin questions because we expected that reasoning about the twins’ brains might be subtle, and we wanted to collect finer-grained judgments than a five-point scale would provide. All questions are included in Supplementary Materials.

### Results and Discussion

#### Standard Assessment for Theory of Intelligence (Three Items)

As in Study 1, we started by averaging each participants’ responses to the three standard TOI assessment items to create their TOI score. Mean scores ranged from 1 to 5 (*M* = 2.799, SD = 1.141) with higher scores indicating a stronger belief that intelligence is fixed. There was no correlation between people’s age and these scores (*R*^2^ = 0.00, *p* = 0.32) nor was there effects of education [*F*(4,58) = 1.64, *p* = 0.177] or gender [*F*(1,61) = 0.003, *p* = 0.96]. There was a marginally significant effect of race [*F*(3,59) = 2.25, *p* = 0.09]. A Tukey test revealed only a significant difference between Asian/Pacific Islander and Caucasian responses [*t*(57) = 5.82, *p* < 0.001] where the two Asian/Pacific Islander participants believed intelligence to be more malleable than the Caucasian participants.

For the analyses treating TOI as categorical, we sorted participants into groups as in Study 1, by separating the responses into terciles: participants with mean scores from 1 to 2.332 were labeled *incremental theorists* (*n* = 31); those with scores from 2.333 to 3.666 were labeled *neutral theorists* (*n* = 11); those with scores from 3.667 to 5 were labeled *entity theorists* (*n* = 22).

#### Essentialism (Eight Items)

We calculated an essentialism score for each person by averaging over the Eight essentialism questions. These scores ranged from 1.37 to 4.5 (*M* = 3.24, SD = 0.618) where higher scores indicate a more essentialized view of intelligence. There was not a significant correlation between age and essentialism scores (*R*^2^ = 0.015, *p* = 0.324) nor was there an effect of gender [*F*(1,61) = 0.279, *p* = 0.599]. There was a small, marginally significant effect of race on essentialism scores [*F*(4,58) = 2.55, *p* = 0.064]. *Post hoc* comparisons revealed the only significant difference to be between the Caucasian participants and the two Asian/Pacific Islander participants [*t*(6) = 3.62, *p* = 0.064] where the Asian/Pacific Islanders saw intelligence as less essentialized than the Caucasians did. There was a small but significant effect of education level on people’s essentialism scores [*F*(4,58) = 2.72, *p* < 0.05]. Where those with “some college, no degree” had slightly more essentialized views of intelligence than those who indicated they “graduated high school” [*t*(15) = 4.29, *p* < 0.01].

##### Coherence of theories of intelligence with essentialism

As in Study 1, participants’ TOI scores were correlated with their responses to the essentialism questions. Treating TOI as continuous, we found a strong correlation between a person’s TOI and the degree of essentialism in thinking about the topic (*R*^2^ = 0.45, *p* < 0.001). The more a person believed intelligence to be fixed, the more essentialism was evident in their reasoning about it. Treating TOI as categorical, there was a main effect of TOI on essentialism responses [*F*(2,60) = 19.83, *p* < 0.001, η^2^ = 0.398]. Tukey contrasts show that incremental theorists rejected essentialist statements more than entity theorists [*t*(47) = 5.97, *p* < 0.001], as did neutral theorists [*t*(28) = 4.43, *p* < 0.001]. There was not a significant difference between neutral theorists and incremental theorists [*t*(26) = 1.58, *p* = 0.377].

#### Predictions about the Brains of Identical Twins Separated at Birth

**FIGURE 4:**
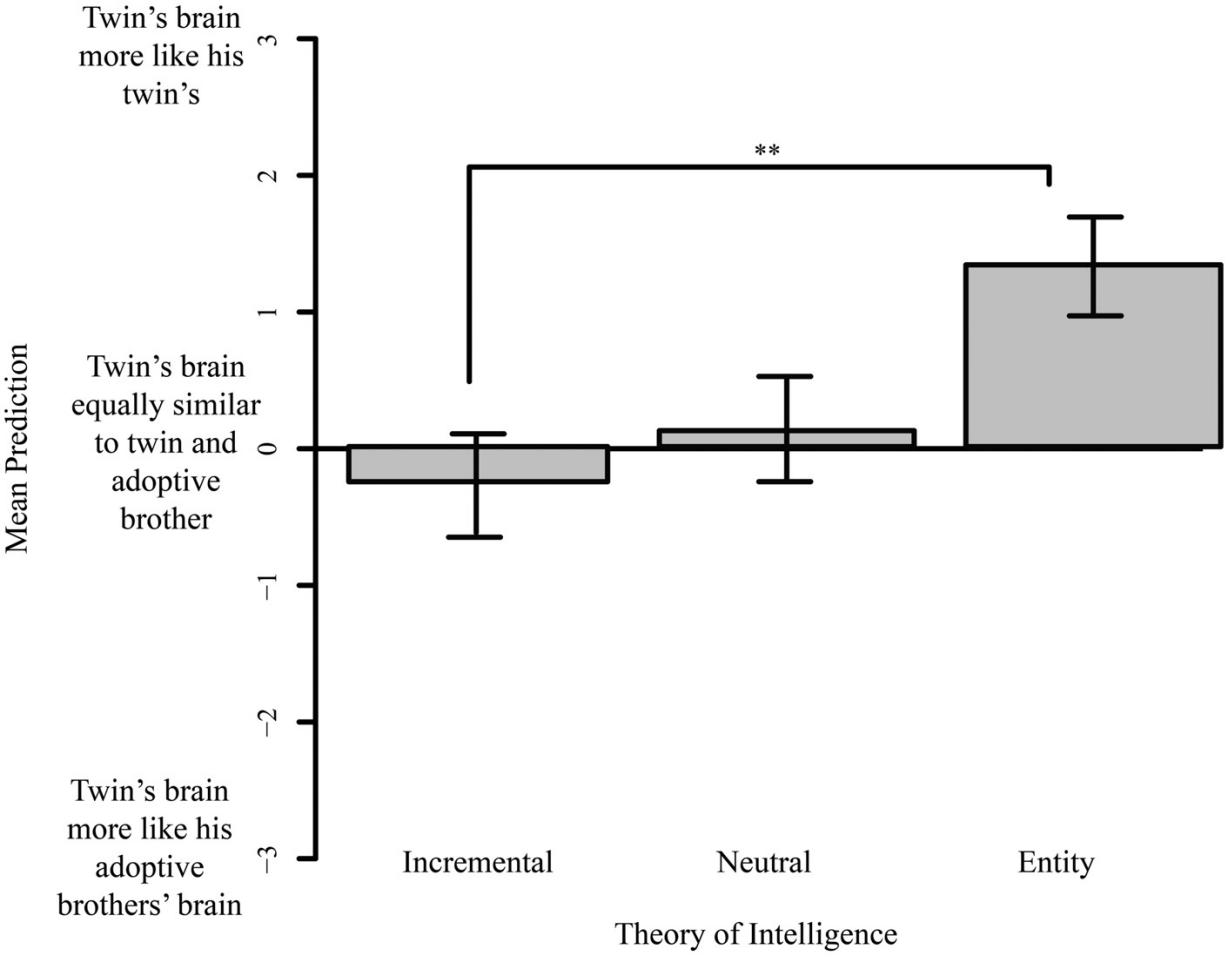
**Biological versus adoptive brothers.** Mean difference in predictions about similarity of biological twins’ versus adoptive brother’s adult brains, with participants grouped by theory of intelligence. Error bars indicate SE. **p < 0.01.

In the following analyses, we compared participants’ intuitions about the similarity between the biological twins’ brains, versus the similarity between the adoptive brothers’ brains (see **Figure 4**).

##### Judgments about brothers in the academic household

Treating TOI as continuous, we found a strong correlation between people’s TOI scores and their judgments about the brains of the identical twins versus the two adoptive brothers raised in the ‘academic’ household (the household where the parents valued and supported academic achievement). The more a person believed intelligence to be fixed, the more similar they predicted the identical twins’ brains would be, compared to their predictions about the similarity of the adoptive brothers (*R*^2^ = 0.23, *p* < 0.001). Treating TOI as categorical, we found that a one-way ANOVA revealed the same pattern [*F*(2,60) = 6.136, *p* < 0.005, η2 = 0.162]. Entity theorists predicted that the biological twins’ brains would be more similar to each other than the brains of the adoptive brothers; incremental and neutral theorists did not make this prediction [entity and incremental *t*(44) = 3.267, *p* < 0.005; entity and neutral *t*(28) = 2.212, *p* < 0.05; neutral and incremental *t*(27) = 0.943, *p* = 1.00].

##### Judgments about brothers in the athletic household

There was a similar correlation between TOI and judgments about the characters in the athletic household (the household where the parents valued and supported achievement in sports). The more a participant believed intelligence to be malleable, the more similar they believed the adoptive brother’s brains would be, relative to the biological twins’ brains (*R*^2^ = 0.122, *p* = 0.004). Thus people who believed intelligence is fixed gave more weight to heredity in brain development; people who believed intelligence is malleable gave more weight to environmental factors. Treating TOI as categorical, we again found a main effect of TOI group on these judgments [*F*(2,60) = 3.51, *p* = 0.0417, η^2^ = 0.100]. Specifically, entity theorists predicted that the brains of the biological twins would be more similar than the brains of the adoptive brothers; incremental and neutral theorists did not share this intuition [entity and incremental *t*(47) = 2.58, *p* < 0.05; entity and neutral *t*(25) = 0.280, *p* = 0.28; neutral and incremental *t*(26) = 0.49, *p* = 1.00].

#### Theories of Intelligence: Categorical or Continuous?

As in Study 1, we were interested in whether people’s TOI (as assessed by the three standard survey items, as well as by our novel items) are best treated as a categorical or a continuous variable. And just as in Study 1, responses to the standard assessment items were bimodally distributed (indicating that people *either* saw intelligence as fixed *or* as malleable), whereas responses to the ‘twins’ questions were not. This is replicates the finding of Study 1, suggesting that although the three standard assessment items give the impression that TOI are categorical, most people actually believe that a combination of genetics and environment affect the brain, and the relative weight they assign to those factors varies continuously along a dimension from emphasizing genetics over environment to emphasizing genetics and environment equally. Note that the judgments are not symmetrical: entity theorists assign genetics a much greater role than incremental and neutral theorists assign to the environment.

Because of the smaller sample size in Study 2, we used Shapiro–Wilk tests to assess these distributions. As in Study 1, participant’s responses on the three standard TOI assessment items were not normally distributed (*W* = 0.89, *p* < 0.001). They fell into a bimodal distribution, with the two modes reflecting ‘entity’ and ‘incremental’ TOI. Also as in Study 1, responses to the essentialism items were normally distributed (*W* = 0.9749, *p* = 0.23). Predictions about the relative similarity of the biological and adoptive brothers were also normally distributed (Academic household *W* = 0.97, *p* = 0.15, Athletic household *W* = 0.97, *p* = 0.12).

## General Discussion

We set out to answer two questions about TOI. The first was whether TOI are coherent and broader than the three questions typically used to assess them; the second was whether these theories are best thought of as discrete categories, or as points on a continuum.

### Coherence and Breadth of Theories of Intelligence

The word ‘theory’ implies a rich, structured set of beliefs that allow a person to reason about a particular domain. But TOI are most often assessed using the same three survey items (*Your intelligence is something about you that you can’t change very much; You have a certain amount of intelligence and you can’t do much to change it; You can learn new things, but you can’t really change your basic intelligence).* It seems important to establish that the TOI that researchers have identified are not merely an artifact of those three survey items, but are rather a coherent set of beliefs that extend beyond those particular statements, to reasoning about intelligence and the brain in general. In the present study, we compared people’s responses on the three standard assessment items to their responses on a broader range of questions about intelligence and the brain. These included eight items probing the essentialism in participants’ beliefs (Studies 1 and 2); 26 items about the brain (Study 1) and three items about the similarity between pairs of biological versus adoptive siblings (Study 2).

In each case, we found that participants’ responses on the three standard assessment questions were strongly correlated with their responses on the other item types. This was true for Study 1 where participants were asked about their beliefs in the abstract. For example, the more a person believed that intelligence is fixed, the more they believed that a person’s brain is fixed (i.e., determined by genetics instead of environment) Likewise, the more a person believed intelligence can change, the more they believed that a person’s brain could change as a result of practice or the environment. We also found a correlation in Study 2, where participants were asked about a more concrete scenario that described twins separated at birth. In other words, participants’ TOI do seem to be coherent, and do extend beyond the three statements typically used to assess them. This sits well with findings showing folk theories to be coherent in other domains (e.g., Vosniadou and Brewer, 1992; Lautrey and Mazens, 2004; Smith, 2007; Shtulman and Schulz, 2008; Shtulman and Calabi, 2012). These findings provide support for the TOI construct, and for the continued use of the three standard assessment items.

### Are Theories of Intelligence Categorical or Continuous?

The second goal of the project was to determine whether TOI represent discrete categories (which are the way they are usually treated in the literature) or a continuous dimension. In other words, is it the case in real life (as in the literature) that most people fall *either* in the entity camp *or* the incremental camp, and not somewhere in between? In the present paper, we explored this in two ways. One was to give participants the three standard assessment items, but to include a neutral answer option so that participants were not forced to choose an answer from one side or the other. Second, we looked at the distribution of responses both for the three standard items, as well as for the broader set of questions about intelligence, brain development and the twins separated at birth. If people’s thinking about the malleability of intelligence naturally falls into two distinct categories, then people’s responses on all of these question types should be bimodally distributed, with responses clustered into two groups, reflecting the two TOI.

The answer to this question turned out to be different for the three standard TOI items than for the other item types. Answers to the three standard TOI items did form a bimodal distribution, even though respondents had the option of the neutral response. However, responses to all of the other item types were normally distributed. In other words, we did not find evidence for two distinct theories in people’s broader beliefs about intelligence and the brain. Instead we found a continuum of beliefs. This was true for abstract questions about essentialism and brain development as well as for more concrete judgments about the twins separated at birth and their adoptive brothers.

## Conclusion

It seems that the three standard items used to assess TOI do tap into a deeper and broader set of coherent beliefs, but that these beliefs exist on a continuum. To be clear, psychologists working with the TOI construct have not argued that entity and incremental theorists are separate and fixed categories of people. On the contrary, many studies have tried to nudge people away from an entity theory and toward an incremental theory. For example, Bergen (1991) had some participants read an essay arguing that traits are malleable, and had others read an essay arguing that traits are fixed. Participants who read the argument for malleability were more likely to attribute failure to situational factors than participants who read the argument for fixedness. Because of results like this, Dweck et al. (1995a) have described implicit TOI as stable, but malleable qualities rather than fixed dispositions. Our data are partially consistent with this characterization: the theories do seem relatively stable and coherent, but our data do not suggest that they are distinct.

The present findings also add to a growing body of work on essentialist reasoning about psychological characteristics. For example, Gelman et al. (2007) found that by third grade, children are more likely to believe that a trait is stable if they also believe it is biologically based or innate. Adults and older children who have essentialist beliefs about one trait tend to have essentialist beliefs about other traits. Gelman et al. (2007) point out that essentialist beliefs might build on each other. For example, a child who thinks that intelligence is fixed would be more likely to agree that intelligence is biologically based and that environment has little effect on a person’s intelligence.

We saw some evidence of this in people’s responses to the different categories of brain-development questions. Entity theorists were more likely believed that intellectual and psychological traits are innate, and reflect genetics and unchanging physical characteristics of the brain. By contrast, incremental theorists were more likely to believe that brain development is affected by practice and by the environment, and that individuals have some control over how their brains develop.

Interestingly, both groups agreed that learning changes the brain—a belief that seems somewhat contradictory for entity theorists. Given that our sample of respondents was relatively highly educated, people may have endorsed the idea that learning changes the brain as a way of expressing their belief in the value of education. Or they may explicitly have been taught (as part of their education) that learning changes the brain. In either case, it would suggest that people’s essentialist reasoning in the domain of intelligence is not 100% ironclad—at least in the case of educated entity theorists.

The current studies add to our understanding of folk TOI, showing that people’s essentialist beliefs are coherent across psychological and physical traits (i.e., judgments about intelligence correspond with judgments about the physical brain) but also that they are not the separate, distinct and self-contained theories that we might imagine them to be. This area of research is one where, for reasons of clarity of thought or exposition, we have imposed a categorical structure on what is actually a continuous dimension. Researchers (including us) find it convenient to talk about ‘entity theorists’ and ‘incremental theorists,’ when in fact most people are somewhere in between. Describing TOI as discrete categories has undoubtedly made for clearer and more engaging stories, while sacrificing some scientific precision. Going forward, it is worth thinking about when to treat TOI as categorical and when to treat them as continuous. For example, when we (as researchers) are trying to give the most accurate possible description of folk beliefs, we should acknowledge that these beliefs exist on a continuum. But when we (as teachers and translators of science) need to simplify the story for a broader audience, the easy-to-understand, categorical story about entity theorists and incremental theorists—while not, strictly speaking the most accurate—may still be the best.

## Conflict of Interest Statement

The authors declare that the research was conducted in the absence of any commercial or financial relationships that could be construed as a potential conflict of interest.
