# Supplementary material for: Exploring the relation between people’s theories of intelligence and beliefs about brain development
Source: Front Psychol. 2015 Jul 3;6:921. doi: 10.3389/fpsyg.2015.00921 (PMC4490215; doi:10.3389/fpsyg.2015.00921)
Supplement: Supplementary file 1 [file Data_Sheet_1.DOCX]

# Study 1: Brain, Mind and Intelligence, Survey

# All questions, except demographic questions were presented in a random order.

**Demographic Questions**

1. What is your age?

_________________________________________________

#### What is the highest level of education you have completed?

( ) 12th grade or less

( ) Graduated high school or equivalent

( ) Some college, no degree

( ) Associate degree

( ) Bachelor's degree

( ) Some graduate school

( ) graduate degree

#### What is your gender?

( ) Male

( ) Female

( ) Other

#### What is your race?

( ) Asian/Pacific Islander

( ) Black/African-American

( ) Caucasian

( ) Hispanic

( ) Native American/Alaska Native

( ) Other/Multi-Racial

( ) Decline to Respond

**Questions to test whether participants were paying attention:**

#### This is a test question please answer 'Strongly Agree'

( ) Strongly disagree ( ) Disagree ( ) Neutral ( ) Agree ( ) Strongly agree

#### There are consistent differences between the brains of aliens and humans. Please answer 'disagree'.

( ) Strongly disagree ( ) Disagree ( ) Neutral ( ) Agree ( ) Strongly agree

#### This is just a test, please answer neutral.*

( ) Strongly disagree ( ) Disagree ( ) Neutral ( ) Agree ( ) Strongly agree

#### This question is just a test. Please select "strongly disagree".*

( ) Strongly disagree ( ) Disagree ( ) Neutral ( ) Agree ( ) Strongly agree

**Theory of Intelligence Sorting Questions**

#### Your intelligence is something about you that you can't change very much.

( ) Strongly disagree ( ) Disagree ( ) Neutral ( ) Agree ( ) Strongly agree

#### You have a certain amount of intelligence and you can't do much to change it.

( ) Strongly disagree ( ) Disagree ( ) Neutral ( ) Agree ( ) Strongly agree

#### You can learn new things, but you can't really change your basic intelligence.

( ) Strongly disagree ( ) Disagree ( ) Neutral ( ) Agree ( ) Strongly agree

**Essentialism Questions**

#### It is easy to change a person's intelligence. It is not a fixed attribute of the person

( ) Strongly disagree ( ) Disagree ( ) Neutral ( ) Agree ( ) Strongly agree

#### Being intelligent has broad ramifications: it influences people's behavior in a wide variety of situations and in many aspects of their lives.

( ) Strongly disagree ( ) Disagree ( ) Neutral ( ) Agree ( ) Strongly agree

#### People who have intelligence will tend to display it in a consistent manner, showing it in different situations and with different people.

( ) Strongly disagree ( ) Disagree ( ) Neutral ( ) Agree ( ) Strongly agree

1. To what extent can people change whether they are intelligent if they want to?

( ) Strongly disagree ( ) Disagree ( ) Neutral ( ) Agree ( ) Strongly agree

#### Intelligence is a deeply-rooted part of the personality: it lies deep within the person and underlies the person’s behavior.

( ) Strongly disagree ( ) Disagree ( ) Neutral ( ) Agree ( ) Strongly agree

#### It will be possible in the future, for scientists to determine a person's intelligence by examining his or her DNA.

( ) Strongly disagree ( ) Disagree ( ) Neutral ( ) Agree ( ) Strongly agree

#### People are born with a predisposition to be intelligent.

( ) Strongly disagree ( ) Disagree ( ) Neutral ( ) Agree ( ) Strongly agree

#### People either have intelligence or they do not: those who have it are a distinct type of person.

( ) Strongly disagree ( ) Disagree ( ) Neutral ( ) Agree ( ) Strongly agree

**Brain Questions**

Learning Questions

#### Learning a new skill makes a lasting change in the brain.

( ) Strongly disagree ( ) Disagree ( ) Neutral ( ) Agree ( ) Strongly agree

#### There is NO change in a person's brain after he or she learns to ride a bike.

( ) Strongly disagree ( ) Disagree ( ) Neutral ( ) Agree ( ) Strongly agree

#### A person's brain DOES NOT change after he or she learns how to swim.

( ) Strongly disagree

( ) Disagree

( ) Neutral

( ) Agree

( ) Strongly agree

#### There is a lasting change in a person's brain after he or she learns a new language.

( ) Strongly disagree ( ) Disagree ( ) Neutral ( ) Agree ( ) Strongly agree

#### Learning is due to modifications in the brain.

( ) Strongly disagree ( ) Disagree ( ) Neutral ( ) Agree ( ) Strongly agree

Innateness Questions

#### The characteristics of a person's brain at birth is the largest determining factor in whether or not that person will be considered a genius later in life.

( ) Strongly disagree ( ) Disagree ( ) Neutral ( ) Agree ( ) Strongly agree

#### To what extent are the characteristics of a person's brain determined by genetics?

( ) No effect ( ) Minimal effect ( ) Modest effect ( ) Strong effect ( ) Very strong effect

#### To what extent do the characteristics of a person's brain at birth determine how early he or she is able to read?

( ) No effect ( ) Modest effect ( ) Moderate effect ( ) Strong effect ( ) Very strong effect

#### To what extent do characteristics of a person's brain at birth determine whether or not he or she is able to learn calculus later.

( ) No effect ( ) Minimal effect ( ) Modest effect ( ) Strong effect ( ) Very strong effect

#### If we had the technology, you could determine a person's adult intelligence by examining his or her brain at birth.

Willful Control

#### People can change the characteristics of their brain if they want to.

( ) Strongly disagree ( ) Disagree ( ) Neutral ( ) Agree ( ) Strongly agree

#### A person has control over his or her brain.

( ) Strongly disagree ( ) Disagree ( ) Neutral ( ) Agree ( ) Strongly agree

Environment

#### Depending on environment, a child's brain will develop in different ways.

( ) Strongly disagree ( ) Disagree ( ) Neutral ( ) Agree ( ) Strongly agree

#### Environment is the largest determining factor in brain development.

( ) Strongly disagree ( ) Disagree ( ) Neutral ( ) Agree ( ) Strongly agree

#### A person's brain is continually changing because people experience new things.

( ) Strongly disagree ( ) Disagree ( ) Neutral ( ) Agree ( ) Strongly agree

Practicing Questions

#### If a person practices speaking a new language, his or her brain will change as a result.

( ) Strongly disagree ( ) Disagree ( ) Neutral ( ) Agree ( ) Strongly agree

#### Is a person's brain is physically changed by practicing an athletic skill, such as shooting a basketball, swinging a golf club or serving a tennis ball?

( ) Definitely No change ( ) Probably no change ( ) Unsure ( ) Probably a change ( ) Definitely a change

#### There is a change in the brain after someone practices lines for a play. [in order to memorize them]

( ) Strongly disagree ( ) Disagree ( ) Neutral ( ) Agree ( ) Strongly agree

#### A person's brain changes after they practice doing a specific type of math problem.

( ) Definitely No change ( ) Probably no change ( ) Unsure ( ) Probably a change ( ) Definitely a change

Brain Basis of Traits Questions

#### The specific characteristics of a person's brain is what causes a person's personality.

( ) Strongly disagree ( ) Disagree ( ) Neutral ( ) Agree ( ) Strongly agree

#### The specific characteristics of a person’s brain has NO effect on whether he or she is able to cope with stressful situations.

( ) Strongly disagree ( ) Disagree ( ) Neutral ( ) Agree ( ) Strongly agree

#### There are not differences between the brains of people who are outgoing and those who are not outgoing.

( ) Strongly disagree ( ) Disagree ( ) Neutral ( ) Agree ( ) Strongly agree

#### There are consistent differences between the brains of people who are good at math and people who are bad at math.

( ) Strongly disagree ( ) Disagree ( ) Neutral ( ) Agree ( ) Strongly agree

#### To what extent do characteristics of a college student's brain affect his or her ability to focus in school?

( ) No effect ( ) Minimal effect ( ) Modest effect ( ) Strong effect ( ) Very strong effect

#### There are consistent differences between the brains of people who are good at drawing realistic scenes and those who are not good at drawing realistic scenes.

( ) Strongly disagree ( ) Disagree ( ) Neutral ( ) Agree ( ) Strongly agree

#### There are consistent differences between the brains of people who are intelligent and the brains of people who are not intelligent.

( ) Strongly disagree ( ) Disagree ( ) Neutral ( ) Agree ( ) Strongly agree

# Study 2: Twin Study

# Note: All questions, except demographic questions were presented in a random order.

**Demographic Questions**

1. What is your age?

_________________________________________________

#### What is the highest level of education you have completed?

( ) 12th grade or less

( ) Graduated high school or equivalent

( ) Some college, no degree

( ) Associate degree

( ) Bachelor's degree

( ) Some graduate school

( ) graduate degree

#### What is your gender?

( ) Male

( ) Female

( ) Other

#### What is your race?

( ) Asian/Pacific Islander

( ) Black/African-American

( ) Caucasian

( ) Hispanic

( ) Native American/Alaska Native

( ) Other/Multi-Racial

( ) Decline to Respond

**Questions to test whether participants were paying attention:**

#### This is a test question please answer 'Strongly Agree'

#### ( ) Strongly disagree ( ) Disagree ( ) Neutral ( ) Agree ( ) Strongly agree

#### To what extent are John and Paul's parents the same? Please put identical

( ) entirely different ( ) almost entirely different ( ) more different than the same ( ) equally similar and dissimilar ( ) more the same than different ( ) close to the same ( ) identical

#### To what extent are Dan and Luke the same. please answer "entirely different"

( ) entirely different ( ) almost entirely different ( ) more different than the same ( ) equally similar and dissimilar ( ) more the same than different ( ) close to the same ( ) identical

#### How intelligent are you? While we care, please put slightly disagree.

( ) Strongly disagree ( ) Moderately disagree ( ) Slightly disagree ( ) Neutral ( ) Slightly agree ( ) Moderately agree ( ) Strongly agree

## Theory of Intelligence Sorting Questions

#### Your intelligence is something about you that you can't change very much.

( ) Strongly disagree ( ) Disagree ( ) Neutral ( ) Agree ( ) Strongly agree

#### You have a certain amount of intelligence and you can't do much to change it.

( ) Strongly disagree ( ) Disagree ( ) Neutral ( ) Agree ( ) Strongly agree

#### You can learn new things, but you can't really change your basic intelligence.

( ) Strongly disagree ( ) Disagree ( ) Neutral ( ) Agree ( ) Strongly agree

**Essentialism Questions**

#### It is easy to change a person's intelligence. It is not a fixed attribute of the person

( ) Strongly disagree ( ) Disagree ( ) Neutral ( ) Agree ( ) Strongly agree

#### Being intelligent has broad ramifications: it influences people's behavior in a wide variety of situations and in many aspects of their lives.

( ) Strongly disagree ( ) Disagree ( ) Neutral ( ) Agree ( ) Strongly agree

#### People who have intelligence will tend to display it in a consistent manner, showing it in different situations and with different people.

( ) Strongly disagree ( ) Disagree ( ) Neutral ( ) Agree ( ) Strongly agree

1. To what extent can people change whether they are intelligent if they want to?

( ) Strongly disagree ( ) Disagree ( ) Neutral ( ) Agree ( ) Strongly agree

#### Intelligence is a deeply-rooted part of the personality: it lies deep within the person and underlies the person’s behavior.

( ) Strongly disagree ( ) Disagree ( ) Neutral ( ) Agree ( ) Strongly agree

#### It will be possible in the future, for scientists to determine a person's intelligence by examining his or her DNA.

( ) Strongly disagree ( ) Disagree ( ) Neutral ( ) Agree ( ) Strongly agree

#### People are born with a predisposition to be intelligent.

( ) Strongly disagree ( ) Disagree ( ) Neutral ( ) Agree ( ) Strongly agree

#### People either have intelligence or they do not: those who have it are a distinct type of person.

( ) Strongly disagree ( ) Disagree ( ) Neutral ( ) Agree ( ) Strongly agree

## Twins Questions

#### 16) At age 25, how different are Scott and Paul's brains? (Scott and Paul are the identical twins and have never met)

( ) Their brains are entirely different (meaning as different as two human brains can be)

( ) Their brains are close to being entirely different

( ) Their brains are more different than the same

( ) Their brains are equally similar and different

( ) Their brains are more similar than different

( ) Their brains are close to the same

( ) Their brains are identical

#### 17) At age 25, how similar are Scott and Dan's brains? (Scott and Dan are adoptive brothers, who grew up in the academic household).

( ) Their brains are entirely different (meaning as different as two human brains can be)

( ) Their brains are close to being entirely different

( ) Their brains are more different than the same

( ) Their brains are equally similar and different

( ) Their brains are more similar than different

( ) Their brains are close to the same

( ) Their brains are identical

#### 18) At age 25, how similar are Paul and Luke's brains? (Paul and Luke are adoptive brothers, who grew up in the athletic household).

( ) Their brains are entirely different (meaning as different as two human brains can be)

( ) Their brains are close to being entirely different

( ) Their brains are more different than the same

( ) Their brains are equally similar and different

( ) Their brains are more similar than different

( ) Their brains are close to the same

( ) Their brains are identical
